# Of Sophists and Spin-Doctors: Industry-Sponsored Ghostwriting and the Crisis of Academic Medicine

**DOI:** 10.4103/0973-1229.58824

**Published:** 2010

**Authors:** Leemon McHenry

**Affiliations:** * *Lecturer in Philosophy, California State University, Northridge, USA.*

**Keywords:** *Academic Medicine*, *Ghostwriting*, *Key Opinion Leader*, *Medical Education and Communication Companies*, *Pharmaceutical Industry*, *Publications Strategy*, *Sophists*

## Abstract

Ghostwriting for medical journals has become a major, but largely invisible, factor contributing to the problem of credibility in academic medicine. In this paper I argue that the pharmaceutical marketing objectives and use of medical communication firms in the production of ghostwritten articles constitute a new form of sophistry. After identifying three distinct types of medical ghostwriting, I survey the known cases of ghostwriting in the literature and explain the harm done to academic medicine and to patients. Finally, I outline steps to address the problem and restore the integrity of the medical literature.

## Introduction

Pharmaceutical companies commonly employ ghostwriters, or uncredited authors, to write or draft manuscripts that subsequently appear in peer-reviewed medical journals under the name of one or more academic researchers. This practice, however, goes beyond simple drafting of a manuscript; it provides an academic façade for research that has been designed, conducted and analyzed by industry and for review articles that similarly obscure the contributions of industry. Such camouflaged authorship undermines scientific integrity and jeopardizes public health.

## Publications Strategy

Early in the process of drug development, a pharmaceutical company maps out a series of pre-clinical and clinical studies, identifying target journals and “key messages” tied to the promotion of the drug. This “publications strategy” is an integral component of drug development and marketing, but problems arise when manuscripts are developed with a focus on marketing goals and with little or no consultation from the eventual ‘authors.’ As a consequence, it is frequently unclear which, if any, published studies represent independent evaluation of a company’s products. Pharmaceutical marketing documents reveal that medical journal articles, letters to the editor and abstracts for professional conferences are designed to give the sales force “tools to drive prescriptions” (Moffatt and Elliott, 2007).

Ghostwritten articles are strategically placed and designed to give the publications the appearance of objectivity when in fact they conceal pervasive conflicts of interest. The whole process of publication planning designed by industry is described by Sergio Sismondo as “ghost management,” of which the ghostwriting is only one component of an invisible process (Sismondo, 2009). The companies use medical writers to prepare manuscripts for publication.

Medical writers work, often as “freelance” contractors, for medical education and communications companies (MECCs), public relations firms or directly for pharmaceutical companies.

Medical writing industry is well established. One published survey identified 182 MECCs operating in the United States in 2001 (Golden *et al*. 2002). Organizations of medical writers in the United States and Europe conduct seminars and conferences, and publish their own professional journals. In addition to the preparation of manuscripts, posters and slides, the MECCs are engaged in promotional activities such as the organization of advisory board meetings with academics to prepare the ‘launch’ of a new drug or a new indication for a drug (e.g., adolescent depression, high cholesterol or social anxiety disorder). MECCs also prepare regulatory submissions, organize continuing medical education (CME) for physicians, dinner meetings, satellite symposia and develop promotional media (CDs, DVDs and websites). Some companies specialize, while others perform multiple functions.

The MECCs or public relations firms charge $18,000 to $40,000 per manuscript, depending on the number of drafts produced and other services specified in the contracts such as organizing teleconferences and advisory meetings. Some of the most frequently used MECCs and public relations firms that produce the manuscripts include: Scientific Therapeutics Information, Inc., Current Medical Directions, Current Medicine Group, Compete Healthcare Communications, Complete Medical Communications Limited, Carus Clinical Communications, Medical Education Systems, DesignWrite, Watermeadow Medical, Envision Pharma, Intramed, Rx Communications, Adis Communications, Xcenda, Excerpta Medica, Adelphi Ltd., and Ruder Finn*.

Scientific Therapeutics Information advertises itself on its website as “a full-service medical publishing group specializing in the development of scientific literature and other resource media with direct application to clinical therapeutics” with a staff that “is intimately familiar with the drug development process and the best possible use of print material to create and sustain awareness for a given concept, drug, or group of drugs, using a fair, balanced approach that maximizes credibility” (STI, 2006). This statement has disappeared from the most recent revision of the company’s advertisement yet the current one still appeals to the promotional mission of their clients (McHenry and Jureidini, 2008; see also STI, 2006).

Among the objectives offered by the MECCs, the most frequently cited include: “increase product market share” and “differentiate product from competitors.”

While there is some blurring of boundaries, industry-sponsored ghostwriting takes at least three forms:

- Ghostwriting for Collaborative Research: One common form of ghostwriting is a technical write-up of clinical research sponsored by a pharmaceutical company in which a medical writer is engaged via a contract with a medical communications company. The named ‘authors’ of the paper are academics or clinical investigators who might contribute to the design of the trial, carry out the collection of data from the sites of the trial, and participate in revisions of the drafts of the manuscript that is produced by the medical writer. While some of these academics qualify for authorship status, others are given purely ‘honorary’ authorship. The medical writer of the drafts disappears from the published paper or is acknowledged in the fine print for “editorial assistance” or “manuscript preparation.” One of the main problems with this type of ghostwriting is misreporting of the data to favor the sponsor company’s product. The control of the message remains with the sponsor company rather than with the named academics on the published paper. This is accomplished through the ghostwriter since the sponsor company supplies summaries of the data that create the appearance of favorable results.
  **Note:** *These names either appear in discussion later, or are well known from litigation.
- Ghostwriting for Paid Honorary Authors: Pharmaceutical companies frequently use in-house medical writers or medical communications companies to produce manuscripts, which are then offered to an ‘honorary’ academic author to affix his or her name before the paper is submitted for publication (Fugh-Berman, 2005). These publications are typically, but not always, review articles. In this type of ghostwriting, the signed ‘author’ has played no role in the research or the writing of the paper, and may or may not revise the paper. The actual writer is not acknowledged at all. Honorary authors are typically ‘key opinion leaders’ – the industry term for academics who are sought by pharmaceutical companies because of their credentials and their ability to influence other prescribers. Key opinion leaders are crucial to the promotional strategy of the company. In the attempt to gain dominance in the market share for a blockbuster drug, many are used to fight ‘competitive issues.’ For example, the companies compensate key opinion leaders to have their names appear on ghostwritten articles and letters that focus on the weakness of competitors’ drugs (McHenry, 2005). A former medical writer explained how a drug company approved the ghostwritten drafts before they were sent to doctors listed as ‘authors.’ Some doctors made no changes at all to the drafts. Others who were not “particularly malleable” were dropped from further projects (Peterson, 2002).
- Ghostwriting Articles on Prescribers’ Experiences with Drugs: Companies also create ghostwriting programs specifically to build product loyalty among prescribing physicians by providing them with opportunities to publish. Pharmaceutical sales representatives visit the prescribing physicians and encourage those who have had favorable experiences with the drugs to liaise with a medical writer to produce a draft of a paper. Many of these publications have appeared in the literature as case studies. One such program called ‘CASPPER’ (Case Study Publications for Peer Review) created in 2000 by SmithKline Beecham sought physicians with positive prescribing experience with the SSRI antidepressant paroxetine who would connect to the medical communication company, Complete Healthcare Communications, in order to expand the database of published data to support paroxetine or help SmithKline Beecham fight competitive issues with other SSRI manufacturers (Perrone, 2009). The acronym’s similarity to the name of a famous cartoon ghost ‘Casper’ was apparently no accident. SmithKline Beecham budgeted for 50 articles under the CASPPER program in 2000 alone (Hill, 2009).

Physicians may rationalize their participation in the publication of ghostwritten articles because they read and agreed with the manuscript, or even because they made a number of editorial changes they believed qualified as authorship. However, this fails to address the main problem that key marketing messages have already been incorporated into the manuscript. More seriously, there may be no one among the academic authors who has analyzed the raw study data. In most cases, if not all, the investigators and the ghostwriter are merely supplied with summaries of the data from the sponsor company.

The medical communications companies that produce the articles take steps to disguise their involvement in the publications. It is common practice for the company to provide a submission package to the ‘lead author’ of the paper including a cover letter to the editor and a submission copy of the paper without the title page identifying the medical communication company and the writer.

Another former medical writer described how medical writing agencies take additional steps to conceal their involvement, including systematically scrubbing electronic documents to remove the names of the medical writing agency, the ghostwriter and the pharmaceutical company (Rees, 2003). Once the ghostwritten pieces are published, they are then cited in the promotional materials of the pharmaceutical companies, and in the peer-reviewed medical literature, as independent verification of the efficacy and safety of a drug. This I have called “the circle of evidence” since marketing is traced right back to marketing instead of independent scientific research (McHenry, 2005). The ghostwritten articles, case studies and letters are considered commercially valuable journal content, which pharmaceutical companies purchase in large quantities for distribution by pharmaceutical sales representatives. Sales of thousands or tens of thousands of article reprints are common.

In the case of a widely-prescribed ‘blockbuster’ drug, reprints can number more than a million. Moreover, some for-profit journals have a business model that depends on the sale of reprints of industry-sponsored articles. The journals themselves produce press releases for newsworthy items thereby creating more reprints orders and revenue for the journal.

Journal supplements are special extra issues of the journal that focus attention on a single drug. These are entirely funded by a pharmaceutical company and typically result from papers delivered at a medical symposium (Lexchin and Light, 2006). Many, if not all, of these articles in the journal supplements are ghostwritten. For just one example of this burgeoning industry, GlaxoSmithKline in 2003 supported the supplement “Advancing the Treatment of Mood and Anxiety Disorders: The First 10 Years’ Experience with Paroxetine” with an “unrestricted educational grant.” The papers that appear in this issue of *Psychopharmacology Bulletin* feature key opinion leaders in psychiatry who discuss the use of paroxetine for everything from depression to social anxiety disorder, generalized anxiety disorder, posttraumatic stress disorder, obsessive-compulsive disorder, premenstrual dysphoric disorder and mood and anxiety disorders in children and adolescents, but there is no statement in this volume that identifies the role of GlaxoSmithKline or Scientific Therapeutics Information in the production and approval of the manuscripts (Nemeroff, 2003).

The reprint and journal supplement revenue combined with the vast revenue from pharmaceutical advertising has created a dangerous dependence between industry and the medical societies that own the journals. Editors from several top medical journals have expressed alarm over these relationships. Richard Horton, editor of *The Lancet*, has written that journals “have evolved into information laundering operations for the pharmaceutical industry” (Horton, 2004). Richard Smith, former editor of the *British Medical Journal* and chief executive of BMJ Publishing, echoes this complaint that medical journals have become a marketing arm of the pharmaceutical industry (Smith, 2005). The financial relationships compromise the credibility of the medical literature because it is widely perceived that journals favor the commercially valuable content produced by pharmaceutical companies over more balanced studies or those that provide a critical evaluation of the studies produced by industry. It is less than clear how many journal editors are seriously concerned about the ghostwritten papers they publish, especially when they do not follow the advice of peer review. They will certainly be aware of the fact that many of the manuscripts that they accept are prepared by the MECCs or industry publication strategy.

Ghostwriting continues as industry finds novel ways to circumvent policies and conceal its inner workings. One reason why ghostwriting has been so difficult to expose to the public view is due to legislation which allows drug and medical device manufacturers to claim that their business practices are protected from revealing secrets to competitors. In the United States, this legislation is called the “Trade Secrets Act.” As a result, only a small fraction of available documents from litigation are released into the public domain.

## Ghostwriting Case Studies: A Window to the Problem

The true extent of ghostwriting for medical journals is unknown, precisely because ghostwriting is meant to be invisible (Ngai *et al*. 2005). An investigation by the United Kingdom House of Commons Health Committee estimated that over 50% of published clinical trials may be ghostwritten (House of Commons, 2005). Much of the public information about the mechanics of ghostwriting is available as a result of legal proceedings against pharmaceutical companies or has been disclosed by physicians who have been approached and refused to participate in such projects. Former-ghostwriters have also contributed to a fuller understanding of the process (Barnett, 2003).

Legal proceedings against drug manufacturers for product liability and fraud reveal behind the scenes organization of publication strategy, contracts with medical communications companies and the participation of key opinion leaders. Some of these cases illustrate how industry control of manuscripts can facilitate manipulation of data to favor the study medication. Others involve illegal marketing on the part of the companies where the ghostwriting is an essential component of the process. The following list updates case studies of ghostwriting identified by Langdon-Neuner in the 2008 issue of *Mens Sana Monographs* (Langdon-Neuner, 2008):

### Gabapentin (Neurontin) -- Sponsorship of scientific articles

A study of documents released in litigation against Parke-Davis, Division of Warner-Lambert Company (subsequently acquired by Pfizer) for off-label promotion of an anti-seizure drug, Neurontin, reveals how Parke-Davis commissioned two medical communication companies, Adelphi Ltd., and Medical Education Systems, to produce a series of articles. Neurontin was initially licensed for adjunctive treatment of partial complex seizures but was being widely prescribed off-label for treatment of pain syndromes and psychiatric conditions for which there was no evidence of efficacy. Documents show that Adelphi and Medical Education Systems planned a series of articles and paid clinicians to work with ghostwriters to produce manuscripts for publications in medical journals on emerging off-label uses of the drug. Steinman *et al*. identified seven published articles that were matched to sponsorship from the medical education company and found that four had favorable conclusions and the other three were neutral (Steinman, 2006).

### Rofecoxib (Vioxx)--Suppressed cardiovascular risks

A study of documents released from litigation against Merck revealed manipulation of data to downplay safety results in the clinical trials of Rofecoxib (Ross *et al*, 2008). In an article published in *Annals of Internal Medicine*, the results of Merck’s “Advantage” trial of Vioxx omitted the deaths of some of the trial participants (Lisse *et al*. 2003). The first author of the paper, Jeffrey Lisse, said: “Merck designed the trial, paid for the trial, ran the trial… Merck came to me after the study was completed and said ‘We want your help to work on the paper.’ The initial paper was written at Merck, and then it was sent to me for editing.” When asked about the death of one woman in the trial who had become the subject of debate inside of Merck, Lisse replied: “Basically, I went with the cardiovascular data that was presented to me (Berenson, 2005).” Vioxx was withdrawn from the market in September of 2004. Graham *et al*. estimate that the drug may have caused up to 120,000 cardiovascular events in the United States, including 40,000 to 60,000 that were fatal (Graham *et al*. 2005).

### Sertraline (Zoloft) –Publications strategy

A study of documents released from litigation against Pfizer’s SSRI antidepressant Zoloft showed that a medical communication company, Current Medical Directions, was preparing, on behalf of Pfizer, 85 papers for publication. 55 of these papers appeared in the leading medical journals between 1998 through 2000 (Healy and Cattell, 2003). In the published articles, there was a consistent emphasis on the positive profile of Zoloft and an under-reporting of side effects. The publications strategy prepared by Current Medical Directions listed studies still in preparation for publication and identified authors for case studies that were already written up as “TBD,” or “to be determined.” [This document can be found at: www.healyprozac.com/GhostlyData/zoloftpublications.htm.]

### Paroxetine (Paxil, Seroxat)–Battle between eli lilly and smithkline beecham over SSRI withdrawal/discontinuation

A study of released documents in a legal case involving failure to warn of withdrawal side effects of the SSRI antidepressant paroxetine showed how SmithKline Beecham engaged the public relations firm, Ruder Finn, to write letters to the editor of *The Journal of Clinical Psychiatry* using key opinion leaders as the ‘authors’ of the letters (McHenry, 2005). Several letters were prepared by ghostwriters at Ruder Finn defending paroxetine under names of different ‘authors.’ The letters were to be sent to the journal in response to campaign launched by Eli Lilly that attacked paroxetine’s severe withdrawal effects compared to fluoxetine (Prozac). One of the letters was published with modifications under the name of Bruce Pollock in *The Journal of Clinical Psychiatry* (Pollock, 1998; for details, see McHenry, 2005). The letter was referenced in SmithKline Beecham’s Business Plan Guide as a “great resource for addressing the issue of discontinuation” and was made available for distribution by the sales force. [See McHenry, 2005. The document can be found at: www.abcnews.go.com/images/Primetime/paxil_bpg.pdf.]

### Paroxetine (Paxil, Seroxat)–Off-label prescribing for adolescent depression

A study of released documents in a legal case involving marketing the SSRI antidepressant paroxetine for adolescent depression revealed that a pivotal clinical trial conducted by SmithKline Beecham, study 329, was ghostwritten by a medical communications company, Scientific Therapeutics Information. The resulting publication by Martin Keller *et al*. in the *Journal of the American Academy of Child and Adolescent Psychiatry* (Keller *et al*. 2001) manipulated the data to make it appear that the trial was a success when, in fact, it failed both requirements for efficacy and safety (Jureidini *et al*. 2008). SmithKline Beecham distributed this study with Medical Query letters to doctors prescribing paroxetine off-label. The study was also cited in the medical literature to demonstrate the effectiveness of paroxetine in treating adolescent depression.

The ghostwriter of this paper produced all drafts from a summary of the Final Study Report and revised with limited input from the named authors (McHenry and Jureidini, 2008). She was acknowledged in the fine print for “editorial assistance.” [Documents can be found at: www.healthyskepticism.org/documents/PaxilStudy329.php]

### Vagus nerve stimulator–Undisclosed background promotion

Device manufacturers also rely on ghostwriters. For example, Charles Nemeroff *et al*. in a 2006 paper published in the journal *Neuropsychopharmacology* wrote in positive terms about a medical device manufactured by the Cyberonics Company (Nemeroff *et al*, 2006). The article, however, fails to disclose the authors’ financial relationship to the company. At the time of publication, Dr. Nemeroff, the lead author of the paper, was editor-in-chief of the journal and the head of the Cyberonics advisory board. An investigation by members of the American College of Neuropsychopharmacology, the journal publisher, accused the lead author of running a “slick public relations disinformation campaign, hiring a ghostwriter, and incestuously placing the article in his own journal (Holden, 2006).”

Cyberonics ordered 10,000 copies of reprints from the journal. A medical writer who worked from materials provided by Cyberonics’ advisory board meetings wrote the first draft of the paper and was acknowledged for editorial support. She claimed that the paper was not ghostwritten and that her role defined her as a ‘facilitator’ (Armstrong, 2006).

### Fen-Phen–Diet drug promotion

Fen-Phen (fenfluramine and phentermine), an anti-obesity drug combination produced by Wyeth-Ayerst Laboratories, was withdrawn from the market in 1997 after reports of valvular heart disease and pulmonary hypertension, primarily in women who had been undergoing treatment with Fen-Phen. According to documents released in litigation, Wyeth launched a public relations campaign that was designed to present obesity as a dangerous health problem to justify the potential risks of Fen-Phen and then a ‘medical education’ campaign that included a ghostwriting strategy for publications. Wyeth hired the medical communications firm, Excerpta Medica, and paid the company $200,000 to draft ten articles for medical journals to promote obesity treatment by presenting the drug in the most favorable light and downplaying any risks. Wyeth’s own clinical trial data had shown only a three per cent difference between Fen-Phen and placebo (Mundy, 2001). Excerpta Medica then paid academic researchers $1,000 to $1,500 to edit the drafts and submit with their names as ‘authors.’ Wyeth maintained control over the content of these articles by editing any material that could damage sales, including the link between the drug and primary pulmonary hypertension (Elliott, 2004).

The publications strategy also included a plan to submit the ghostwritten articles to academic journals owned by the publisher, Reed Elsevier, of which Excerpta Medica happened to be a branch. Only two of these articles were published before Fen-Phen was removed from the market, neither of which disclosed the relationship to Excerpta Medica. [There have been several discussions of this episode in the literature, see especially Elliott, 2004, and Mundy, 2001].

### Prempro

Harmone Replacement Therapy. A court order in another case against Wyeth demanded release of documents that revealed a paper trail of ghostwriting for 18 medical journals, including *The American Journal of Obstetrics and Gynecology* and *The International Journal of Cardiology*. Between 1998 and 2005, Wyeth engaged the medical communication company, DesignWrite, to outline and draft articles that would be ‘authored’ by top physicians. Prempro, a combination of estrogen and progestin, was promoted for women to protect against aging skin, heart disease and dementia until a federal study found that hormone therapy was linked to breast cancer (Singer, 2009).

### Harm to academe

Publications serve as the basis for prestige and advancement among academics. Ghostwriting, however, creates an uneven playing field, allowing some ‘authors’ benefit from the services of invisible scribes. More importantly, the integrity of science depends on the trust placed on individual clinicians and researchers, and on the peer-review system, which is the foundation of a reliable body of knowledge. Academic authorship is an assertion of intellectual responsibility. It is assumed that the signed authors have collectively been responsible for study design, conduct, data analysis and writing. Similarly, a review article is expected to represent the analysis and conclusions of the signed authors. When academics allow their names to appear on ghostwritten articles and letters, they betray this basic responsibility and are guilty of academic dishonesty (Horton, 2004). Such behavior would be considered unacceptable in most academic disciplines, but has become standard practice in medicine.

Since universities have become dependent on the revenue from clinical trials conducted on their campuses, they have turned a blind eye to the embellishment of their academics’ *curriculum vitae* with the ghostwritten publications (McHenry, 2007). While universities typically embrace honor codes that include punitive measures for students who engage in plagiarism (including research papers bought off the internet), faculty who are guilty of submitting ghostwritten work from MECCs are not subject to the same treatment. This creates a serious problem for campus morale and academic leadership.

Honorary and gift authorship are deceptive to the readers of the journal articles, case studies and letters to the editor. The basic problems surrounding these conflicts of interest include:

- the sponsor company’s role in the origin of the publication,
- the true role of the medical writer in the production of drafts and in the interpretation of data,
- the influence of ‘internal’ authors of the sponsor company in their interactions with the medical writer,
- the financial relationship between the medical writer, the medical communication company and the sponsor company, and
- the true roles of the named ‘external’ authors on the publication.

Industry-supported control and suppression of the academic process also occurs when: (1) Pharmaceutical companies threaten to withdraw financial support or educational grants to medical or bioethics organizations that publish critical studies (Elliott, 2001). (2) Medical journals reject studies critical of pharmaceutical marketing, especially when editors follow legal advice to the journal on the basis that they are subject to libel actions. (Healy, 2008) (3) Pharmaceutical companies threaten legal action to the journals that consider publishing critical studies, letters and reviews (McHenry, 2008).

### Harm to patients

There is little doubt that the ghostwritten publications are meant to influence physicians’ prescribing habits. However, the publications have distorted the profile of the drugs by suppression of negative data. First, in most cases only the positive clinical trials are selected for the ghostwritten publications. Many of these trials are designed to favor the study medication (Safer, 2002). Second, ghostwriting can lead to manipulation of results by selective reporting of data within one clinical trial (Chan *et al*. 2004). As the case of Vioxx demonstrates, industry ghostwriting has great potential to cause harm to patients.

Off-label prescriptions involve the use of drugs for conditions other than those approved by the US FDA. An approved drug can be prescribed for any reason at the discretion of a physician. Marketing off-label use is illegal for pharmaceutical companies, but it is not illegal for them to launder their promotional efforts through key opinion leaders who promote off-label use at professional conferences and in the medical journals.

Many of the posters, abstracts and articles for these conferences and journals have been ghostwritten, several of which have distorted results by overstating efficacy and downplaying safety. Ghostwriting has also been used to advance the cause of disease mongering. This occurs as a result of pharmaceutical marketing of indications created specifically to increase prescriptions, such as “social anxiety disorder,” “pediatric bipolar disorder,” “premenstrual dysphoric disorder” and the like (Moynihan and Cassels, 2005). Medical communication companies and public relation firms are engaged to create awareness of a previously unrecognized ‘disease’ and the new treatments for ‘sufferers’ of these conditions. Manuscripts are prepared in the manner described above with key opinion leaders as the ‘authors’ for these publications. As a result of these activities, patients have been prescribed medications in which the potential for harm outweighs the benefit. The pharmaceutical industry has thus been accused of marketing disease for what are merely ordinary conditions of life and of exacerbating unhealthy reliance on and over-use of drugs (House of Commons, 2005).

### Industry response

The pharmaceutical industry has been conspicuously silent about ghostwriting. In one report, however, witnesses from both GlaxoSmithKline and AstraZeneca strongly denied that their respective companies engaged in ghostwriting practices with one industry spokesperson claiming: “The issue of ghost-writing, as alleged, is not something I recognize at all” (House of Commons, 2005). Advertising executives have defended the role of medical communications by arguing that they work with scientists and help direct research toward drugs that patients most desire. They further claim that they “neither toy with science nor ghostwrite articles that physicians use to make decisions about prescribing drugs” (Peterson, 2002). When, however, GSK’s ghostwriting program, CASPPER, was revealed in court documents, the president of one medical communication company, Rockpointe, defended ghostwriting as “common practice” and claimed: “ghostwriting is not only legal, but it is necessary and essential in producing timely information to the public about drugs and devices. Without such a practice, patients, doctors, and science itself would be at a standstill because the timing of such articles would be severely altered without ghostwriting” (Sullivan, 2009).

**Figure 1:**
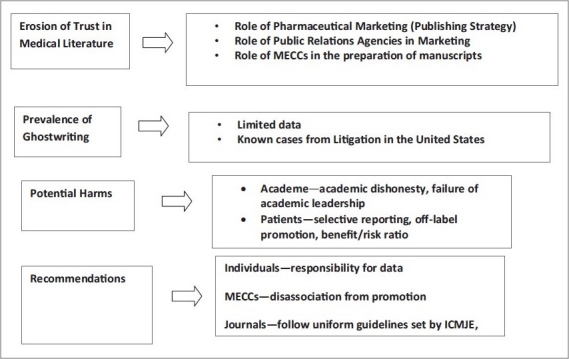
Flowchart of the paper

## Recommendations

To reduce the potential harms from ghostwriting and restore trust in the medical literature, I propose the following:

### Individuals

No clinician or researcher should allow an article to be published that in any way misrepresents his or her contribution to study design, data analysis or manuscript preparation or fails to disclose the intellectual contributions and financial relationships of those involved in preparation of the manuscript. Moreover, no clinician should participate in the publication of an article if he or she has not had full and unrestricted access to the raw data and can be confident that all analyses have been appropriately conducted. Signing a confidentiality agreement with a sponsor company should be the first signal that the scientific status of the study is potentially compromised.

Medical communications companies involved in the communication of scientific results must disassociate themselves from the sponsor company’s promotional activities. Otherwise, they have no serious claim to medical writing as opposed to drug promotion. Companies and individuals in the business of manuscript preparation should obtain contractual assurances that their specific contributions will be fully acknowledged in all publications. Organizations of medical writers such as the European Medical Writers Association (EMWA) and the American Medical Writers Association (AMWA) have established guidelines for medical writing that strongly emphasize transparency and honesty in the reporting of data (Jacobs and Wager, 2005; see also Wager, 2007). These guidelines should be rigorously followed.

Journals. The International Committee of Medical Journal Editors (ICMJE), a small working group of general medical journals, has issued policy for ethical principles in the conduct and reporting of medical research, including guidelines for authorship. This is determined by: substantial contributions to conception and design, or acquisition of data, or interpretation of data; drafting the article or revising it critically for important intellectual content; and final approval of the version to be published (ICMJE, 2001). The World Association of Medical Editors (WAME), another international association of medical journal editors, has established its own statement to deter ghostwriting. WAME insists on full disclosure of all contributions to published research including explicit mention of individuals and affiliations, and all financial sources that produced the research and the writing of the manuscript. WAME also advises editors who discover ghostwriting to report the offense to the authors’ academic institutions and publish a notice that article was ghostwritten along with the names of the responsible companies and named authors (WAME, 2005).

The problem with these recommendations, however, is that they have not been uniformly adopted and ghostwriting persists even in journals that have adopted ICMJE policy. Every medical journal should adopt strict conflict-of-interest policies that clearly establish the intellectual contributions of all signed authors and specifically ask about contributions from individuals who are not listed as authors. Finally, in order for the journals to be fully transparent, they should disclose their own revenue related to sale of reprints, pharmaceutical advertising and journal supplements.

## Conclusion

The sophists of 5^th^ century B. C. Greece were the ancient counterparts to our modern spin-doctors and advertisers. They shaped public opinion by skillful mastery of persuasive speaking. The essence of sophistry is persuasion without regard for any considerations of truth. In fact, the sophists abandoned truth. The traditions of rationality upon which science rests challenged the sophistical methods of persuasion, but the sophistry did not die in the 5^th^ century B.C. It continues to thrive with new purpose and technique. In our age, the rise of pharmaceutical marketing is a new form of sophistry that undermines the integrity of the research published in the peer-reviewed medical journals and the reputation of the medical profession. The serious attempt to discover efficacy or safety in medicine is irrelevant to the goal of promotion.

### Take home message

As the modern sophists have infiltrated the medical journals and made this their forum for promotion, medical rhetoric has usurped medical science—an embarrassment in an age allegedly devoted to evidence-based medicine. It is therefore imperative that medicine reclaim ground loss to the profit motive of industry.

### Conflict of interest

Leemon McHenry has been research consultant to the law firm of Baum, Hedlund, Aristei & Goldman, Los Angeles, California, since 2003.

### Declaration

An earlier version of this paper was presented at a symposium on Science, Communication and Policy, University of Strathclyde, Glasgow, Scotland, in 2009. This is my original unpublished work, of which I hold the copyright, and it has not been submitted for publication elsewhere. I take full responsibility for the veracity of its contents.

## Questions That This Paper Raises

1. How does ghostwriting constitute a new form of sophistry? Have medical journals been usurped by the marketing interests of pharmaceutical and medical device industries?
2. How does the pharmaceutical and medical device industry disguise ghostwriting?
3. Why is so little known about the prevalence of ghostwriting?
4. What is the proper role of journal editors with respect to publishing ghostwritten clinical research and review articles? Are journal editors equally guilty of financial conflict of interest?
5. How does ghostwriting harm academe and patients?
6. What is the industry response to allegations of corrupting the medical literature?

## About the Author


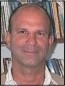
 *Leemon McHenry read philosophy for his PhD at the University of Edinburgh, Scotland. In 2009, he was visiting professor of philosophy and fellow at the Institute for Advanced Studies in the Humanities, University of Edinburgh. His philosophical research interests include the metaphysics of time and the problem of consciousness. In medical ethics he has criticized the corporate take-over of medicine and the corrupting influence of the pharmaceutical industry on medicine, including dubious claims about chemical imbalance as a marketing ploy for selling antidepressants, industry-sponsored clinical research, ghostwriting for medical journals and direct-to-consumer advertising of pharmaceuticals. In a broader realm, he has argued that the industry-academic partnerships have worsened university research, created increased opportunities for scientific misconduct, and have failed to protect academic freedom. He currently divides his time between teaching logic and philosophy of science at California State University and doing research in medical ethics for the law firm of Baum, Hedlund, Aristei & Goldman.*
